# Crystal Ball Health Policies: A Case Against Preventive Testing For Alzheimer’s Disease

**DOI:** 10.3389/fnagi.2022.842629

**Published:** 2022-02-15

**Authors:** Silvia Inglese, Andrea Lavazza, Carlo Abbate

**Affiliations:** ^1^Fondazione IRCCS Ca’ Granda Ospedale Maggiore Policlinico, Geriatric Unit, Milan, Italy; ^2^Centro Universitario Internazionale, Arezzo, Italy; ^3^University of Pavia, Pavia, Italy; ^4^Fondazione Don Carlo Gnocchi Onlus (IRCCS), Milan, Italy

**Keywords:** predictive medicine, biomarkers, genetics, Aduhelm, paternalism

## Abstract

After the recent approval of a new drug for the treatment of Alzheimer’s disease, the first in almost twenty years, it is useful to consider what are the real possibilities to make a preclinical diagnosis of dementia and to treat its symptoms. The scientific community widely agrees that the drugs available today can only slow down the progression of the disease; it, therefore, seems helpful to warn against encouraging the spread of preventive testing. In fact, faced with the prospect of drugs that promise to act in the first stage of Alzheimer’s, there might be an incentive to invest in the research on biomarkers and even healthy adults could be encouraged to increasingly resort to such prediction tests. Our claim, however, is that such massive use of biomarkers would eventually make things worse for many individuals and for society as well. A few examples are given to illustrate this risk. Therefore, our proposal is to limit access to prediction testing until truly effective treatments for Alzheimer’s are available.

## Introduction

The controversy over the FDA’s approval of Aduhelm has put the spotlight back on drug supervision and research around Alzheimer’s disease (Karlawish and Grill, 2021). Aduhelm is based on a monoclonal antibody (aducanumab) that has shown solid results in clearing/eliminating amyloid protein plaques in patients’ brains in the two trials carried out to test its efficacy (EMERGE and ENGAGE; Haeberlein et al., 2019, 2020). However, this result did not translate into a clear improvement in cognitive faculties and daily functioning.

In the uncertainty that still surrounds the clinical efficacy of Aduhelm, the first one approved in the last 18 years, attention must be kept on the so-called biomarkers that may allow an early diagnosis. The availability of a treatment that can potentially slow down the course of the disease in its early stages might increase scientific efforts and investment in finding reliable proxies for the onset of the disease and the predisposition to develop it. Even in these cases, considerations of public health policies and ethical issues are as relevant as strictly biomedical ones. Early diagnosis is a very important clinical objective. In general, in medicine, the earlier the diagnosis, the more effective the treatment. If a genetic predisposition to developing the disease can be identified, as in the case of BRCA1 gene mutations for breast cancer (Peto et al., 1999), early prevention is not only possible but necessary.

### Early Preclinical and Clinical Diagnosis of Alzheimer’s Disease

In the case of Alzheimer’s disease, the situation is more complex from a clinical point of view as well as from a bioethical and public health policy perspective. Firstly, currently available drugs for AD (e.g., donepezil, rivastigmine, galantamine, and memantine) do not stop or reverse dementia, but at most slow it down. In this regard, aducanumab seems no different. Indeed, regulatory bodies have established that any new dementia drug can only be accepted as effective if it slows the symptoms’ progression, reducing the rate of cognitive and/or functional deterioration caused by the disease over time (European Medicines Agency, 2018). This criterion of efficacy, which can be called “soft”, was adopted considering that degeneration is a progressive, incurable disease, whereby slowing it down is considered an acceptable benefit.

Secondly, extensive research efforts in recent years have identified multiple modifiable risk factors for dementia, i.e., less education, hypertension, hearing impairment, smoking, obesity, depression, physical inactivity, diabetes, infrequent social contact, excessive alcohol consumption, head injury, and air pollution (Livingston et al., 2020). However, it is estimated that modifying these 12 risk factors might prevent or delay no more than 40% of dementia cases (Livingston et al., 2020). Furthermore, the first RCTs carried out on healthy older adults at risk of dementia (FINGER, MAPT, PreDIVA) aimed to verify the effectiveness of prevention programs found improvements in cognitive functioning, but not always a consequent decrease in the incidence of future cases of dementia (Ngandu et al., 2015; van Charante et al., 2016; Andrieu et al., 2017; Rosenberg et al., 2020).

Thirdly, one should consider the extreme length of the so-called preclinical phase of the disease (Dubois et al., 2016). In particular, it has been estimated that pathological changes in the two key players in the pathogenesis of AD, i.e., the beta and tau proteins, begin up to 20 and 15 years before the onset of symptoms, respectively (Bateman et al., 2012; Buchhave et al., 2012; Fagan et al., 2014).

Finally, a complication is related to the genetics of AD. Research has found that genes play a strong role in Alzheimer’s disease (AD), with late-onset AD (LOAD) showing 58–79% heritability and early-onset AD (EOAD) exceeding 90% (Sims et al., 2020). In line with this finding, twin studies have predicted the heritability of late-onset forms to be as high as 80% (Bettens et al., 2013). LOAD has an onset starting at age 65 and is the most common form of AD accounting for approximately 95% of all cases. EOAD has an onset before age 65 and accounts for up to 5% of all cases. The amyloid precursor protein (APP), presenilin 1 (PSEN1), or presenilin 2 (PSEN2) genes were the first to be identified primarily in EOAD (Bettens et al., 2013; Sims et al., 2020). In parallel, a form of the apolipoprotein E gene (i.e., the APOE ε4 allele) is probably the best understood genetic factor in LOAD. More recently, large-scale association studies have found that more than ten risk genes for late-onset disease and more than 50 loci are implicated in AD (Bettens et al., 2013; Sims et al., 2020).

Although many genes are involved in AD, only rarely has a simple genetic change been found to directly cause the disease. In fact, there are some families with EOAD (familial-EOAD) in which mutations in one of three genes (i.e., APP, PSEN1, or PSEN2) are transmitted between successive generations by autosomal dominant inheritance and virtually guarantee that a person who inherits one of them will develop AD (Bettens et al., 2013; Sims et al., 2020). But these mutations account for less than 1% of people with Alzheimer’s disease.

In most cases, the genetic mechanisms of Alzheimer’s remain largely unexplained, and the genetic factors are likely to be complex. For example, about 25% to 30% of the population carries the APOE ε4 allele, which increases the risk of Alzheimer’s disease in a dose-dependent way. In fact, people with one ε4 allele have about a three-fold increased risk of Alzheimer’s disease, and those with two ε4 alleles have about a 15-fold increased risk, compared with those with the most common genotype, APOE ε3ε3 (Bettens et al., 2013; Sims et al., 2020). But not everyone with this gene variation develops the disease. In summary, to date, we cannot identify any simple genetic modification that allows us to predict disease onset before symptom onset in most cases of AD.

Despite this scenario of soft efficacy of available treatments, lack of certainty on prevention, an extremely long preclinical phase, and complex genetic background, early diagnosis of AD in the clinical phase of the disease, when symptoms have already started, is highly 1recommended (Alcove Project, 2017; Bianchetti et al., 2019). Indeed, multiple benefits of early diagnosis of AD have been recognized, from implementing early interventions including cognitive stimulation and rehabilitation, more accurate coordinated therapeutic plans, better management of patient symptoms and safety, lower health care costs, and later institutionalization (Alcove Project, 2017; Bianchetti et al., 2019).

The problem we want to discuss here originates instead with the prospect of an early diagnosis of AD in the *pre*clinical phase of the disease. This goal now seems to be getting closer given the continuous progress in the identification and validation of simple and rapid disease biomarkers, which can now also be found in blood (Hampel et al., 2018; Molinuevo et al., 2018; Nakamura et al., 2018; Schindler et al., 2019; Karikari et al., 2020), that seem to be able to identify or predict disease onset many years before the onset of symptoms. At this point, we would like to consider some issues that are related to these diagnostic opportunities. If, in general, earlier is always better, in these specific cases some relevant questions arise.

### Discussion: Ethical Issues Related to the Preclinical Diagnosis of Alzheimer’s Disease

It is obviously not a question of individuals resorting to this type of test: this falls within the autonomy of the patient who gives explicit consent based on correct information. In this regard, in a recent survey by Alzheimer’s Research UK, 74% of people responded that they would want to know if they had Alzheimer’s disease before symptoms develop (Alzheimer’s Research UK, 2019). However, it is not unlikely that some individuals, knowing that they may develop AD in the future without having absolute certainty, will have more to lose than to gain from the test result, in terms of serious psychological and existential consequences. In this sense, it should also be considered that not all people have the psychological, cultural, and social resources to understand and manage a diagnosis of that kind (Harzheim et al., 2020). Some degrees of paternalism might be recommended here, as we explain below.

While the benefit of prevention of modifiable risk factors for AD remains uncertain, the anguish caused by trying to ward off the disease and seeing the first symptoms of memory appear can take such a heavy toll on the person that it can affect their whole life. The same can happen in the opposite direction: the belief that one has little time left to live fully can lead to renouncing long-term plans, focusing on maximum satisfaction in the present moment driven by fear of the future. All this seems highly dysfunctional in the face of an advantage that for now seems uncertain and limited considering the actual possibilities of prevention and treatment.

On the other hand, it could be argued that having greater knowledge of the future that awaits us, even if it is negative and marked by illness, is a way of having greater awareness and control over one’s life. Knowledge is an end in itself and this is particularly true of self-knowledge. In this sense, from an ethical viewpoint, it would be questionable to prevent individuals from having access to this kind of predictive testing if there was a clear and consistent motivation accompanied by an understanding of the consequences that such knowledge might cause.

But one should also take into account a well-known psychological effect. Regardless of one’s situation in absolute terms, what matters for psychological well-being is the relative direction of one’s life, whether it is upward or downward. A person who has a large fortune but sees their wealth slowly eroding will experience increasing frustration, while a person who has good career prospects and has obtained a salary increase will feel joy and see their life as promising even if they are still almost in poverty because of the debts incurred to pay for their degree. Therefore, knowing that there is a likelihood of developing Alzheimer’s in the future will set a sad tone for one’s entire existence even if one is living a good and fulfilling life.

This ethical debate related to AD is still in its infancy, but a similar case was discussed in the late 70s about predictive tests for Huntington’s chorea. At that time physicians were on different sides when it came to resorting to clinical prediction. “The medical profession is shown to be deeply divided on the ethics of a predictive test for Huntington’s chorea. Some members are already using the prospect of a reliable test as an inducement to potential transmitters of this incurable hereditary disease to postpone procreation. Other members would prefer to see any future test withheld from every applicant until such time as radically improved means of treatment or a cure is discovered” (Thomas, 1982).

Besides that issue, an even more pressing one is the medicalization of one’s whole life and the stigma attached to a disease with devastating consequences on the cognitive sphere that does not currently appear to have a secure chance of being delayed, stabilized, or cured. One might then ask whether it makes sense to invest in research into predictive biomarkers when there is no treatment available with this kind of efficacy. But this consideration would be short-sighted. The study to eradicate a growing threat to the peaceful aging of millions of people must proceed on all fronts and seek to exploit all possibilities of advancing knowledge and capacity to intervene to reduce the patients’ distress.

Rather, our take on the topic is that, in the absence of effective treatments that can help prevent or at least delay the onset of symptoms, if not stop or reverse the disease, predictive tests should not be commercialized or used in a generalized way in clinical practice. The motivation for this health policy direction stems from the likely social effects of large-scale dissemination of predictive tests in the absence of progress in treatment and prevention tools (Fulda and Lykens, 2006).

One can object that people could still take the test and keep the result secret. But it is probable that individuals will be induced to implement a series of behavioral choices, in the hope of improving their preventive diagnosis, that will still not give them adequate or complete protection from AD and would potentially reveal the diagnosis to others. This may include a special diet, a lifestyle aimed at continuous cognitive stimulation, taking supplements or medication (Rothstein, 2020).

Indeed, there are obvious situations in which the diagnosis could not be held back in any case. This would be the case in the selection of personnel for professions or posts requiring above-average mental efficiency, or in the selection of candidates for important political posts. In fact, once a test for a more or less reliable predictive diagnosis of Alzheimer’s disease is available and widespread, public opinion will demand that any future senator or governor show evidence of long-term cognitive efficiency. And if the politician shows susceptibility to dementia, even in the not-too-distant future, their career would probably be doomed.

But does it make sense to lose an excellent candidate today just because they could be struck down by a debilitating form of Alzheimer’s in 10- or 20-years time? Would it not mean unnecessarily giving up an asset for the whole of society? Of course, it is possible to argue that in 10- or 20-years time, we may find ourselves with a leader whom we consider to be experienced and in excellent condition, but who is beginning to develop some minor symptoms, not publicly disclosed, but which can affect the leader’s performance when they are called upon to make important choices. In that case, we might perhaps regret not having demanded that they undergo predictive testing.

But if we were to administer biomarkers or other tests for AD earlier and earlier, even if they were accurate and could predict the onset of the disease with a good percentage (reliability is not at issue here), what would be the benefit to us individually and socially? Who will marry someone who in the near or distant future is likely to lose their memory, not recognize their spouse and no longer be self-sufficient? Who will want to have a child with an individual who has a high probability of being an incapable and absent parent, perhaps at the most sensitive age of the child’s life?

And who will hire an experienced middle-aged worker to play a major role in their company if they do not want to take the predictive test for Alzheimer’s or test positive for it? And which company will still want to ensure an individual who may soon need expensive long-term care? Certainly, legislative remedies could be introduced, whereby no discrimination should be applied to those who are likely, to whatever degree, to develop some form of dementia.

But even such interventions to avoid negative spillovers related to predictive testing cannot undo the negative effects of large-scale use, including a possible inefficient allocation of resources for biomedical research (Juth and Munthe, 2012). Indeed, those who received a positive diagnosis with respect to the possibility of developing Alzheimer’s disease will inevitably push for society to invest as much money as possible to achieve an effective cure.

Depending on the balance between politicians and influential people who tested positive or negative, AD research would become the priority, absorbing a lot of funding at the expense of equally important research on other diseases, which might end up receiving less attention.

Ultimately, the greatest risk would be a social segmentation that could lead to a kind of marginalization of those known to have tested positive for Alzheimer’s disease. They would end up becoming a *de facto* stigmatized group with reduced opportunities compared to the rest of the population, based on their alleged destiny to fall ill and plunge into the nightmare of losing the memory of their identity.

If this scenario seems excessively pessimistic and unrealistic, it is certainly useful to underline the potentially problematic and controversial aspects linked to the diffusion of biomarkers or other types of screening able to indicate the possibility of developing Alzheimer’s disease with good probability and considerable advance.

It is not a matter of stopping the research focused on preventing AD, but of assessing the social impact that the dissemination of new, more or less reliable, predictive clinical tools could have. In the absence of a truly effective therapy, able to postpone the onset, stop or reverse cognitive impairment, the advantages of knowing about one’s potential predisposition to suffer from dementia may not outweigh the negative effects on an individual and collective level that we have briefly presented in this article. Bioethical reflection and cost-benefit calculations in terms of public health policies should probably take these dynamics into account so that the management of biomedical advances is oriented towards balanced goals.

## Conclusion

Banning or severely restricting something is always a choice to be weighed with the utmost care. The proposal we are putting to the public debate is to continue research into predictive testing for AD but to restrict its application in the absence of a proven preventive treatment or cure capable of stabilizing or curing the disease. This restriction would take the form of non-commercialization and limited access in the clinical setting, with strict protocols and choices left to physicians or even ethics committees in healthcare facilities. In addition, privacy criteria would have to be as restrictive as possible, including all those who undergo screening to avoid discrimination in both negative and positive ways (a candidate for public office could take the test, make it public and then challenge their competitors to do the same).

It is perhaps premature to raise alarms about biomarkers or other types of screening that are not yet available in such advanced forms. Yet the controversial emergency approval of Aduhelm is a reminder that caution is of the utmost importance.

## Data Availability Statement

The original contributions presented in the study are included in the article, further inquiries can be directed to the corresponding author.

## Author Contributions

The authors contributed equally to the manuscript. All authors contributed to the article and approved the submitted version.

## Conflict of Interest

The authors declare that the research was conducted in the absence of any commercial or financial relationships that could be construed as a potential conflict of interest.

## Publisher’s Note

All claims expressed in this article are solely those of the authors and do not necessarily represent those of their affiliated organizations, or those of the publisher, the editors and the reviewers. Any product that may be evaluated in this article, or claim that may be made by its manufacturer, is not guaranteed or endorsed by the publisher.
